# Hypertension Treatment Modality and Suicide Risk Among Veterans in Veterans Health Administration Care From 2015 to 2017

**DOI:** 10.1001/jamanetworkopen.2020.20330

**Published:** 2020-10-12

**Authors:** Kallisse R. Dent, Cameron A. Griffin, John F. McCarthy, Ira R. Katz

**Affiliations:** 1 Veterans Affairs (VA) Serious Mental Illness Treatment Resource and Evaluation Center, Office of Mental Health and Suicide Prevention, Ann Arbor, Michigan; 2 VA Office of Mental Health and Suicide Prevention, Washington, DC

## Abstract

**Importance:**

The Veterans Health Administration (VHA) serves a population of veterans with a high prevalence of comorbid health conditions and increased risk for suicide.

**Objective:**

To replicate the findings of a previous study and assess whether exposure to angiotensin receptor blockers (ARBs) is associated with differential suicide risk compared with angiotensin-converting enzyme inhibitors (ACEIs) among veterans receiving VHA care.

**Design, Setting, and Participants:**

This nested case-control design included all suicide decedents from 2015 to 2017 with a VHA inpatient or outpatient encounter in the prior year and with either an active ACEI or ARB prescription in the 100 days prior to death. Using a 4:1 ratio, controls were matched to cases by age, sex, and hypertension and diabetes diagnoses. Controls were alive at the time of the death of the matched case, had a VHA encounter within the previous year, and had either an active ACEI or ARB medication fill within 100 days before the death of the matched case.

**Exposures:**

An active ACEI or ARB prescription within 100 days before the death of the case.

**Main Outcomes and Measures:**

Cases were suicide decedents from 2015 to 2017 per National Death Index search results included in the Veteran Affairs/Department of Defense Mortality Data Repository.

**Results:**

Among 1309 cases, the median (interquartile range [IQR]) age was 68 (60-76) years and among 5217 controls, the median (IQR) age was 67 (60-76) years, and 1.9% of veterans in both groups were female. ARBs were received by 20.2% of controls and 19.6% of cases; ACEIs were received by 79.8% of controls and 80.4% of cases. The crude suicide odds ratio for ARBs vs ACEIs was 0.966 (95% CI, 0.828-1.127). Controlling for covariates, the adjusted odds ratio for ARBs was 0.985 (95% CI, 0.834-1.164). Sensitivity analyses using only those covariates that differed significantly between groups, restricting to veterans ages 65 and older, dropping matching criteria, and adjusting for the quantity and temporal proximity of ACEI and ARB exposure in the 100 days prior to the index date, had consistent findings.

**Conclusions and Relevance:**

This case-control study did not identify differences in suicide risk by receipt of ARBs vs ACEIs in analyses specific to veterans receiving VHA care in contrast with findings from the referent study.

## Key Points

**Question:**

Are findings from a 2019 study suggesting elevated suicide risk among those receiving angiotensin receptor blocker (ARB) vs angiotensin converting enzyme inhibitor (ACEI) medications replicable in a population of veterans in the Veterans Health Administration (VHA)?

**Findings:**

In this case-control study, 1309 veteran VHA user suicide decedents from 2015 to 2017 were compared with 5217 controls. No significant difference in suicide risk between those receiving ARB vs ACEI prescriptions was found.

**Meaning:**

Results suggest that previous findings of a higher suicide risk among ARB vs ACEI recipients are not generalizable to the population of veterans receiving VHA care.

## Teaser

This case-control study evaluated whether exposure to angiotensin receptor blockers is associated with differential suicide risk compared with exposure to angiotensin converting enzyme inhibitors among veterans in Veterans Health Administration care.

## Introduction

Replication studies can validate or contradict initial observations, evaluate whether findings generalize to other populations, and prompt refinement of explanatory paradigms.^1^ This validation or contradiction is particularly important for complex, multifactorial outcomes such as suicide. Since Durkheim’s groundbreaking study in 1897,^2^ suicide has been investigated within many disciplines and in recent years the volume of empirical suicide studies has increased rapidly.^3^

In 2019, Mamdani and colleagues^4^ observed in an Ontario population that use of angiotensin receptor blockers (ARBs) was associated with greater suicide risk than use of angiotensin converting enzyme inhibitors (ACEIs). ARBs and ACEIs are commonly prescribed to treat hypertension, and these findings were shared widely within the scientific community.^5^ However, the findings regarding ACEIs and ARBs and mental health outcomes and suicide are inconsistent. ACEIs have been found to have both positive and negative effects on depression and no effect on suicide risk.^6,7,8,9,10^ ARB receipt is associated with reduced risks of bipolar disorder, Alzheimer disease, anxiety, and depression.^10,11,12,13,14^ Two studies, 1 based on small counts,^10^ the other by Mamdani and colleagues,^4^ report that ARBs are associated with increased suicide risk.

ACEIs and ARBs may affect mental health outcomes through the renin angiotensin system (RAS), which produces angiotensin II and regulates blood pressure. ARBs decrease blood pressure by preventing angiotensin II from binding to the angiotensin II type 1 (AT1) receptors that are responsible for vasoconstriction of blood vessels in hypertensive patients.^15^ ACEIs inhibit production of angiotensin II, limiting the amount of circulating angiotensin II that can bind to AT1 receptors. Although RAS is responsible for regulating blood pressure peripherally, there is also evidence that tissue RAS, angiotensin II, and AT1 receptors are found in the brain, specifically in regions responsible for hormone and autonomic regulation, sensory perception, and emotional behaviors.^16,17^ Several RAS polymorphisms have positive associations with suicide risk, suggesting that the brain RAS and the medications that act on the system may play a role in suicide behavior.^7,17,18,19^

The biological pathways between ARBs, brain RAS tissue, and suicide behavior are less well understood. ARBs are lipophilic, allowing them to cross the blood-brain barrier, and they block angiotensin II binding to the AT1 receptor.^15,20^ Blocking of the AT1 receptor, and neurotoxicity, is the mechanism cited by many studies that find mental health improvements following ARB receipt.^11,12,13,14^ ACEIs decrease angiotensin II production, also resulting in less binding to AT1 receptors, which could have a similar effect in this pathway. Mamdani and colleagues^4^ described an alternative pathway between ARBs and suicide risk through activation of the hypothalamic pituitary-axis. ARB blocking of the AT1 receptor leads to greater circulation of angiotensin II in the brain, which increases levels of substance P in brain tissue.^21,22^ Higher concentrations of substance P are correlated with hyperactivity of the hypothalamic pituitary-axis, which is indicative of a heightened stress response and is common among patients diagnosed with depression.^23^ In the same pathway, ACEIs would have the opposite effect, decreasing angiotensin II production and dysregulation of the hypothalamic pituitary-axis. Though unclear, these plausible mechanisms between hypertension medications and suicide behavior may encourage additional investigation of this association, especially among high-risk groups.

Surveillance work documents that in the US age and sex adjusted suicide rates for veterans are 1.5 times those of nonveteran US adults.^24^ Suicide prevention is a clinical priority of the US Department of Veterans Affairs (VA). The VA health system, the Veterans Health Administration (VHA), also serves an older population (age ≥65 years) with a high prevalence of hypertension.^25^ Replication of the association between hypertension medications and suicide risk, observed by Mamdani and colleagues,^4^ could have implications for VHA prescribing patterns. Prior to making operational decisions based on the findings of Mamdani et al,^4^ evaluation of whether these findings are also observed in the VHA veteran population is warranted. The aim of this study was to evaluate whether exposure to ARBs is associated with differential suicide risk compared with ACEIs among veterans in VHA care.

## Methods

### Cohort

Replicating the nested case-control design used by Mamdani and colleagues,^4^ the study cohort included all veteran suicide decedents from 2015 to 2017, with a VHA inpatient or outpatient encounter within the year before death and with either an active ACEI or ARB medication fill at the time of death.^4^ Active medications included any inpatient or outpatient VHA prescription that overlapped with the 100 days before the case’s death. Analyses excluded individuals who received both medication types in this period. This work was conducted as part of ongoing VHA operations and program evaluation and was approved by the Veteran Affairs Office of Mental Health and Suicide Prevention and is not classified as research and is therefore exempt from formal review by an institutional review board. This study followed the Strengthening the Reporting of Observational Studies in Epidemiology (STROBE) guidelines for case-control studies.

Before obtaining all eligible controls, we used a power calculation to confirm that the 1311 suicide decedents initially identified in VHA records from 2015 to 2017 were sufficient to make reliable conclusions at the .05 significance level. Power for 2 independent proportions was calculated using the observed relative risk of Mamdani and colleagues,^4^ the estimated exposure of ARB use among the controls, and assuming 4:1 matching. The lower bound of the adjusted effect size (1.33) reported by Mamdani and colleagues^4^ was used as the relative risk. We estimated the relative exposure of ARBs among controls by calculating the mean exposure of ARBs for nonsuicide decedents with either ACEI or ARB use in the 100 days prior to 20 randomly selected dates from 2015 to 2017.

Eligible controls included any veteran alive at the beginning of 2015 with VHA use between January 1, 2014, and December 31, 2017, and an active ACEI or ARB prescription between 2015 and 2017. Controls were matched to cases with the same sex, status of hypertension, and diabetes diagnoses from 2014 to 2017, and age (within 1 year) on the case’s date of death. Each case’s set of eligible controls was restricted to those alive, with VHA use in the previous year, and with an active ACEI or ARB prescription within 100 days before the case’s death. Four eligible controls were randomly selected for each case. Multiple cases could have the same control, but controls were used only once for each case. Controls could later become cases.

### Data Sources

Clinical data, including diagnoses, prescriptions, VHA use, and demographic characteristics were drawn from the Corporate Data Warehouse, which is the comprehensive electronic medical record of the VHA. Mortality was assessed using the National Death Index search results included in the Veteran Affairs and Department of Defense Mortality Data Repository. History of self-harm was also assessed using the Suicide Prevention Application database and the Suicide Behavior Overdose Report, which are used by VHA Suicide Prevention Coordinators to document and track veteran suicide-related behavior.

### Covariates

All covariates presented by Mamdani and colleagues^4^ were analyzed within the VHA cohort: age, sex, urban residence, Charlson Comorbidity Index score, long-term residential care, psychiatry and cardiology appointments, any history of deliberate self-harm, diagnosis of alcohol use disorder, anxiety or sleep disorder, affective disorder, psychoses, other mental health disorder, stroke, chronic kidney disease, chronic liver disease, heart failure, coronary artery disease, hypertension, diabetes, and active prescriptions all based on the preceding year.^4^ Income was not included, due to insufficient data in the VHA Corporate Data Warehouse.

The Charlson Comorbidity Index score used only inpatient diagnoses made in the previous year and was broken into 4 distinct categories: (1) no hospitalizations; (2) at least 1 hospitalization and no relevant Charlson comorbidity diagnoses; (3) at least 1 hospitalization and a Charlson Comorbidity index score equal to 1; and (4) at least 1 hospitalization and a Charlson comorbidity index score greater than or equal to 2. These methods are consistent with the methods used by Mamdani and colleagues.^4^ Sensitivity analyses that created a VHA-specific model used a Charlson Comorbidity Index Score that also included outpatient diagnoses. Long-term residential care was defined as any VHA inpatient specialty lasting more than 90 days in the year before the case’s date of death. Psychiatry and cardiology appointment variables counted the number of days a veteran had at least 1 inpatient or outpatient specialty-specific VHA encounter in the preceding year. Diagnosis variables were assessed on all VHA inpatient and outpatient encounters in the prior year, and *International Classification of Diseases, Ninth Revision (ICD-9)* and *International Statistical Classification of Diseases and Related Health Problems, Tenth Revision (ICD-10)* definitions can be found in eTable 1 in the Supplement. Active prescriptions in the previous year were defined as any prescription overlapping with the year before the veteran’s index date and included the following drug classes: antidepressants, antipsychotics, benzodiazepines, β-adrenergic antagonists, mood stabilizers (lithium, divalproex, valproic acid, oxcarbazepine, and carbamazepine), calcium-channel blockers, cholesterol-lowering medications (fibrins, statins, and resins), potassium-sparing diuretics, loop diuretics, thiazide diuretics, and other antihypertensives. Also included was the unique number of prescription types, from these categories, that a patient had in the prior year.

### Statistical Analysis

Descriptive statistics were calculated and compared across case and control groups using standardized differences. Aligning with the methods used by Mamdani and colleagues,^4^ differences greater than 0.1 indicated a significant difference between the groups. The percentage of cases and controls with ACEI vs ARB exposure in the 100 days prior to the cases death was determined, and conditional logistic regression was used to calculate the odds of suicide given ARB vs ACEI exposure. A multivariable conditional logistic regression model adjusted for the covariates suggested by Mamdani and colleagues^4^ including the Charlson Comorbidity Index score, long-term residential care, psychiatry and cardiology appointments, diagnosis of alcohol use disorder, anxiety or sleep disorder, affective disorder, psychoses, other mental health disorder, stroke, chronic kidney disease, chronic liver disease, heart failure, coronary artery disease, and active prescriptions all based on the preceding year.^4^ Wald 95% CIs were used to determine statistical significance of odds ratios with those excluding 0 indicating a statistically significant result. All analyses were conducted using SAS Enterprise Guide version 8.2 (64-bit) statistical software (SAS Institute).

### Sensitivity Analysis

We conducted several sensitivity analyses to test the robustness of study findings. First, to parallel the Mamdani et al^4^ study population, analyses were conducted in a VHA cohort restricted to patients age 65 years and older.^4^ Second, multivariable conditional logistic regression was conducted using only those covariates that had a standardized difference greater than 0.1 across the case and control groups. This sensitivity analysis replicated the covariate selection methods of Mamdani and colleagues,^4^ rather than using the same variables included in their model, regardless of the covariates standardized differences found in the present study. Third, to determine whether population differences in matching variables in the VHA case cohort contributed to the results, the same analyses were conducted without matching on diabetes, hypertension, sex, or age. Fourth, to account for potential differences in the veterans’ quantity of and temporal proximity to ACEI and ARB exposure, we adjusted for the number of medication coverage days in the 100 days before the index date and the number of days since the veterans’ last active ACEI or ARB medication fill day on the index date.

## Results

### Cohort Creation and Power Calculation

From 2015 to 2017, there were 1311 veteran VHA user suicide decedents with VHA use in the previous year and with either an active ACEI or ARB medication fill in the 100 days prior to their death.

Across 20 randomly selected index dates between 2015 and 2017, the estimated relative exposure of ARBs among the eligible controls was 22.0%. Using this relative exposure, we confirmed the study power to be greater than 0.95 at the lower bound (1.33) of the effect size in the Mamdani et al^4^ study. The 1311 cases identified between 2015 and 2017 provided enough power to detect the expected association between ARBs vs ACEIs and suicide.

Of 1310 cases, a successful match was made to 5238 controls. Each case matched to 4 controls, except for 2; 1 had 3 eligible controls and the other had 2. Nineteen controls became cases. There were 18 veterans in the study cohort, including 1 case, that had missing rurality data. These were excluded and the final analytic cohort included 1309 cases and 5217 controls.

### Descriptive Statistics

Among 1309 cases, the median (interquartile range [IQR]) age was 68 (60-76) years and among 5217 controls, the median (IQR) age was 67 (60-76) years, and 1.9% of veterans in both groups were female. Descriptive statistics for all covariates used in the adjusted analyses of the Mamdani et al^4^ study, are presented in Table 1. Covariates that significantly differed between the case and control groups included diagnosis of alcohol use disorder (17.8% vs 7.7%; standardized difference, 0.31), anxiety or sleep disorder (39.1% vs 24.4%; standardized difference, 0.32), affective disorder (35.7% vs 13.9%; standardized difference, 0.52), psychoses (4.2% vs 2.5%; standardized difference, 0.10), and other mental health disorders in the prior year (27.9% vs 15.0%; standardized difference, 0.32), Charlson Comorbidity Index score in the previous year (for example, including only inpatient diagnoses and a score of 0, 9.7% vs 3.2%; standardized difference, 0.27), antidepressant (52.2% vs 29.1%; standardized difference, 0.48), antipsychotic (17.7% vs 8.0%; standardized difference, 0.29), benzodiazepine (28.3% vs 10.2%; standardized difference, 0.47), mood stabilizer (33.1% vs 20.3%; standardized difference, 0.29), and other hypertensive prescriptions in the prior year (31.4% vs 24.7%; standardized difference, 0.15), the number of psychiatry visits (median [IQR], 0 (0-1) vs 0 (0-0); standardized difference, 0.36), and unique prescription types in the prior year (median [IQR], 4 (2-6) vs 3 (1-4); standardized difference, 0.44), and any history of self-harm (9.5% vs 1.7%; standardized difference, 0.34).

**Table 1.** Descriptive Statistics Across 2015-2017 VHA Suicide Decedents and Matched Controls

Variable	No. (%)^a^		Standardized difference^b^
	Cases (n = 1309)	Controls (n = 5217)	
Age, median (IQR), y	68 (60-76)	67 (60-76)	0.03
Female	25 (1.9)	100 (1.9)	0.01
Charlson comorbidity index score (only including inpatient diagnoses)			
No hospitalizations	992 (75.8)	4482 (85.9)	−0.26
0	127 (9.7)	167 (3.2)	0.27
1	64 (4.9)	96 (1.8)	0.17
≥2	126 (9.6)	472 (9.1)	0.02
Charlson comorbidity index score (including inpatient and outpatient diagnoses)			
0	576 (44.0)	2682 (51.4)	−0.15
1	284 (21.7)	884 (16.9)	0.12
≥2	449 (34.3)	1651 (31.7)	0.06
Visits, median (IQR), No.			
To psychiatrist	0 (0-1)	0 (0-0)	0.36
To cardiologist	0 (0-0)	0 (0-0)	0.04
Residence in long-term care facility	9 (0.7)	30 (0.6)	0.01
Urban residence	769 (58.8)	3253 (62.4)	−0.07
Any history of self harm	124 (9.5)	90 (1.7)	0.34
VHA diagnoses made in the previous year			
Anxiety or sleep disorder	512 (39.1)	1273 (24.4)	0.32
Affective disorder	467 (35.7)	723 (13.9)	0.52
Psychoses	55 (4.2)	129 (2.5)	0.10
Other mental health disorder	365 (27.9)	781 (15.0)	0.32
Alcohol use disorder	233 (17.8)	402 (7.7)	0.31
Heart failure	253 (19.3)	1210 (23.2)	−0.09
Coronary artery disease	261 (19.9)	1063 (20.4)	−0.01
Stroke	82 (6.3)	361 (6.9)	−0.03
Chronic liver disease	60 (4.6)	207 (4.0)	0.03
Chronic kidney disease	148 (11.3)	632 (12.1)	−0.03
Hypertension	1227 (93.7)	4890 (93.7)	0.00
Diabetes	582 (44.6)	2316 (44.4)	0.00
Active prescriptions in the prior year			
Unique, median (IQR), No.	4 (2-6)	3 (1-4)	0.44
Antidepressant	683 (52.2)	1516 (29.1)	0.48
Antipsychotic	231 (17.7)	418 (8.0)	0.29
Benzodiazepine	370 (28.3)	531 (10.2)	0.47
β-adrenergic antagonists	619 (47.3)	2301 (44.1)	0.06
Mood stabilizer	433 (33.1)	1058 (20.3)	0.29
Calcium-channel blocker	413 (31.6)	1666 (31.9)	−0.01
Cholesterol-lowering medication	853 (65.2)	3498 (67.1)	−0.04
Other antihypertensives	725 (55.4)	2711 (52.0)	0.07
Potassium-sparing diuretics	74 (5.7)	322 (6.2)	−0.02
Loop diuretics	234 (17.9)	1004 (19.2)	−0.04
Thiazide diuretics	250 (19.1)	1055 (20.2)	−0.03
Other	411 (31.4)	1290 (24.7)	0.15
ACEI and ARB medication exposure in the 100 d before index date, median (IQR)			
Medication days, No.	88 (61-101)	87 (60-101)	0.03
Days since last medication day on index date, No.	0 (0-3)	0 (0-5)	−0.05

Abbreviations: ARB, angiotensin receptor blockers; ACEI, angiotensin converting enzyme inhibitors; IQR, interquartile range; VHA, Veterans Health Administration.

^a^ No. with exposure for categorical variables. Total No. for continuous variables is presented in the first row.

^b^ An absolute standardized difference greater than 0.1 indicates a significant difference between case and control groups.

### Conditional Logistic Regression

Among cases, 19.63% had ARB exposure in the 100 days before death and 20.22% of controls had ARB exposure (Table 2). At a 95% significance level, conditional logistic regression suggested that the suicide risk among veterans receiving an ARB prescription was not more than 12.7% (odds ratio [OR], 0.966; 95% CI, 0.828-1.127) higher than those receiving an ACEI prescription (Table 3). Adjusting for potential confounders further attenuates the effect (OR, 0.985; 95% CI, 0.834-1.164).

**Table 2.** Exposure of Angiotensin Receptor Blockers and Angiotensin Converting Enzyme Inhibitors Among Veterans Health Administration 2015-2017 Suicide Decedents and Matched Controls^a^

Variable	Patients exposed, No. (%)	
	Suicide decedents (n = 1309)	Controls (n = 5217)
Angiotensin receptor blockers	257 (19.63)	11055 (20.22)
Angiotensin-converting enzyme inhibitors	1052 (80.37)	4162 (79.78)

^a^ The exposure of hypertension medication types among 1309 suicide descendants and 5217 controls matched on age (±1 year), sex, and hypertension and diabetes diagnosis in the prior year.

**Table 3.** Odds of Suicide Among Those With Angiotensin Receptor Blockers vs Angiotensin Converting Enzyme Inhibitors Prescriptions in the 100 Days Prior to the Cases Death Among 2015-2017 Veterans Health Administration Users

Exposure	Odds ratio (95% CI)	
	Unadjusted	Adjusted^a^
Angiotensin receptor blockers	0.966 (0.828-1.127)	0.985 (0.834-1.164)
Angiotensin-converting enzyme inhibitors	1 [Reference]	1 [Reference]

^a^ Multivariable conditional logistic regression adjusted for Charlson Comorbidity Index score (calculated only using inpatient diagnoses), long-term residential care, psychiatry and cardiology appointments, diagnosis of alcohol use disorder, anxiety or sleep disorder, affective disorder, psychoses, other mental health disorder, stroke, chronic kidney disease, chronic liver disease, heart failure, coronary artery disease, and active prescriptions presented in Table 1, all based on the preceding year.

All sensitivity analyses were consistent with these findings (eTable 2 in the Supplement). Limiting to veterans age 65 years and older, the crude effect was not statistically significant (OR, 0.942; 95% CI, 0.783-1.133). Excluding all matching criteria also resulted in a nonstatistically significant effect (OR, 0.863; 95% CI, 0.741-1.005). Including only covariates with a standardized difference greater than 0.1 attenuated the crude OR to a similar magnitude as the full list of covariates included in the adjusted analysis of Mamdani et al^4^ (OR, 0.985; 95% CI, 0.835-1.161).^4^ Adjusting for the quantity and temporal proximity of ACEI or ARB exposure in the 100 days before the veterans’ index date also attenuated the nonstatistically significant effect (OR, 0.987; 95% CI, 0.836-1.166).

## Discussion

Using a nested case-control study among 1309 veteran VHA user suicide decedents between 2015 and 2017, the suicide risk among veterans with an ARB prescription did not significantly differ from those with an ACEI prescription.

In comparison, using administrative claims databases in Ontario, Canada, Mamdani and colleagues^4^ documented the odds of suicide among older patients receiving ARBs as 1.63 (95% CI, 1.33-2.00) times that of those receiving ACEIs, after adjusting for potential confounders. As described by Katz,^26^ these findings indicate a serious but rare adverse drug effect whose replication may help synthesize scientific evidence and inform potential suicide prevention policies. Replicating the Mamdani et al^4^ findings in a VHA Veteran population, we found that the results were not significant (adjusted OR, 0.985; 95% CI, 0.834-1.164), suggesting that the Mamdani et al^4^ findings do not generalize to the VHA population and may not be suitable for informing VHA policy.

These contradictory findings highlight the importance of replication within the field of suicide prevention. The difference in findings may represent differences in the underlying populations or prescribing practices between the Ontario Canada cohort and the VHA cohort. For example, the elevated suicide risk among ARB users in Canada may reflect selection bias, in which there is selective use of ARBs among patients with a greater risk for suicide instead of a pharmacological effect. In contrast, selective use of ARBs among veteran VHA users at a lower suicide risk may obscure a real pharmacological effect. Other common biases that make assessing associations between hypertension medications and suicide complex, include information bias and residual confounding.^27^

### Limitations

This study has limitations. First, chemical and pharmacological diversity occurs within the ACEI and ARB drug classes. For instance, ARBs vary in lipophilicity, making some more likely to cross the blood-brain barrier than others.^15,20^ Individual drug effects on suicide risk may have been diluted by pooling into drug classes. Second, veterans were only required to have an ACEI or ARB exposure at some point in the 100 days prior to their index date. This exposure variable does not consider prescription dose, frequency, or adherence. It also represents an acute exposure, which does not distinguish between veterans with brief and long-term administration of ARBs. Studies suggest that long-term administration of ARBs may increase transport of ARBs across the blood-brain barrier, allowing them to have greater effect on brain function.^20^ Future analyses should follow patients over a longer period to determine if the length and quantity of ACEI or ARB exposure affects suicide risk. Third, this study was limited to veterans with ARB or ACEI exposure and does not compare with those without hypertension medication use. Because both ACEIs and ARBs target the RAS, which plays an active role in stress and inflammatory pathways in the brain, comparison with an unexposed patient population may be warranted in future analyses.^16^ Fourth, there may be residual confounding by socioeconomic status, untreated depression, and other factors associated with adherence to and effectiveness of ACEI and ARB prescriptions.

## Conclusions

This nested case-control study found no association between ARB vs ACEI use and suicide risk among veterans engaged in VHA care. Results did not replicate those of the Mamdani et al^4^ study. These findings do not support modifications in hypertension treatment guidelines in VHA at this time. Furthermore, this conclusion may encourage the development of replication infrastructures that have the capacity to efficiently and effectively replicate suicide literature. The VHA’s comprehensive network of clinical and suicide-related data and the development of a dedicated suicide surveillance expertise may enable replication studies to evaluate potential modifications to VHA treatment protocols. These processes may help to ensure that VHA policies and practices are informed by relevant studies conducted both within and outside of the VHA health system.

## Notes

Supplement. **eTable 1.** ICD9 and ICD10 Diagnosis Codes **eTable 2.** Sensitivity Analyses: Odds of Suicide among those with Angiotensin Receptor Blockers (ARBs) versus Angiotensin Converting Enzyme Inhibitors Prescriptions (ACEIs)
