# Supplementary material for: Hypertension Treatment Modality and Suicide Risk Among Veterans in Veterans Health Administration Care From 2015 to 2017
Source: JAMA Netw Open. 2020 Oct 12;3(10):e2020330. doi: 10.1001/jamanetworkopen.2020.20330 (PMC7550968; doi:10.1001/jamanetworkopen.2020.20330)
Supplement: Supplement. — eTable 1. ICD9 and ICD10 Diagnosis Codes eTable 2. Sensitivity Analyses: Odds of Suicide among those with Angiotensin Receptor Blockers (ARBs) versus Angiotensin Converting Enzyme Inhibitors Prescriptions (ACEIs) [file jamanetwopen-e2020330-s001.pdf]

Supplemental Online Content

Dent KR, Griffin CA, McCarthy JF, Katz IR. Hypertension treatment modality and suicide risk among veterans in Veterans Health Administration care from 2015 to 2017. *JAMA Netw Open*. 2020;3(10):e2020330. doi:10.1001/jamanetworkopen.2020.20330

eTable 1. ICD9 and ICD10 Diagnosis Codes

eTable 2. Sensitivity Analyses: Odds of Suicide among those with Angiotensin Receptor Blockers (ARBs) versus Angiotensin Converting Enzyme Inhibitors Prescriptions (ACEIs)

This supplemental material has been provided by the authors to give readers additional information about their work.

© 2020 Dent KR et al. *JAMA Network Open.*

eTable 1. ICD9 and ICD10 Diagnosis Codes

Covariate[1]	ICD9 Codes	ICD10 Codes
Anxiety or Sleep Disorder	293.84, 300.00, 300.01, 300.02, 300.09, 300.10, 300.20, 300.21, 300.22, 300.23, 300.29, 300.3, 300.7, 327, 780.5, 307.4, 291.92, 347.0, 292.85,	F06.4, F40., F41, F42, F45.20, F45.21, F45.29, G47, F51, F10.182, F10.282, F10.982, F11.182, F11.282, F11.982, F13.182, F13.282, F13.982, F14.182, F14.282, F14.982, F15.182, F15.282, F15.982, F19.182, F19.282, F19.982
Affective Disorder	296, 298.0, 301.1, 300.4, 309.0, 309.1, 311	F3, F06.31, F06.32
Psychoses	295, 296, 297, 298, 299.1	F20, F22, F23, F24, F25, F28, F29
Other Mental Health Disorder[2]	290.8, 290.9, 291, 292 (except 292.82), 293, 294.0, 294.9, 300, 301, 302, 303, 304, 305 (except 305.1)	F01, F02, F06, F10, F11, F12, F14, F15, F16, F18, F19, F20-29, F30-39, F40-49 (except F48.2, F48.8, and F48.9), F50, F53, F60, F63, F68.1, F68.8, F69, F90, F91, R45.7
Alcohol Use Disorder	291, 303, 305.0, 357.5, 425.5, 535.3, 571.1, 571.2, 571.3, 760.71	E24.4 F10, G31.2, G72.1, I42.6, K29.2, K70, K85.2, K86.0
Heart Failure	398.91, 402.11, 402.91, 404.11, 404.13, 404.91, 404.93, 428,	I09.9, I11.0, I13, I25.5, I42.0, I42.0, I42.5, I42.6, I42.7, I42.8, I42.9, I43, I50, P20.0
Coronary Artery Disease	414.0	I25
Stroke	430, 431, 432, 433, 434, 435, 436, 362.34	I60, I61, I62, I63, I65, I66, I67, I68, I97.810, I97.811, I97.820, I97.821
Chronic Liver Disease	571	K70.0, K70.2, K73, K75.4, K75.8, K75.9, K76.0, B18
Chronic Kidney Disease	585	N18, N19
Hypertension	401	I10
Diabetes	250, 357.2, 362.0, 366.41	E10, E11, E13

[1]All diagnosis covariates are mutually exclusive.

[2]All ICD codes used to pull mental health disorders are listed for simplicity, however if any diagnosis was also identified as a psychoses, anxiety, sleep, affective, or alcohol use disorder diagnosis then it was excluded from the 'Other Mental Health Disorder' category.

ICD9 and ICD10 codes used to define diagnosis variables. Listed are the least granular parent code; all children codes under the parent are included in the definition.

© 2020 Dent KR et al. *JAMA Network Open.*

eTable 2. Sensitivity Analyses: Odds of Suicide among those with Angiotensin Receptor Blockers (ARBs) versus Angiotensin Converting Enzyme Inhibitors Prescriptions (ACEIs)

Exposure	Crude OR (95% CI) No Matching[1]	Crude OR (95% CI) Age Restricted, 65+[2]	Adjusted OR (95% CI) Select Variables[3]	Adjusted OR (95% CI) ACEI ARB Exposure
**ARBs**	0.863 (0.741, 1.005)	0.942 (0.783, 1.133)	0.985 (0.835, 1.161)	0.987 (0.836, 1.166)
**ACEIs**	1.0 (reference)	1.0 (reference)	1.0 (reference)	1.0 (reference)

[1]Conditional logistic regression among a case-control cohort without matching on sex, age, or hypertension and diabetes diagnoses. This sensitivity analysis assesses whether population differences in matching variables in the VHA case cohort contributed to our unexpected results.

[2]Conditional logistic regression among a VHA cohort restricted to patients 65 and older, paralleling Mamdani et al.'s study population. All other methods were consistent with methods presented in the primary analysis.

[3]Multivariable conditional logistic regression adjusting for confounders that had a standardized difference greater than 0.1 across the matched case and control groups among our 2015-2017 VHA user cohort. Adjustment variables included diagnosis of alcohol use disorder, anxiety or sleep disorder, affective disorder, psychoses, and other mental health disorders in the prior year, Charlson Comorbidity Index score in the prior year (calculated using both inpatient and outpatient diagnoses), anti-depressant, antipsychotic, benzodiazepine, mood stabilizer, and other hypertensive prescriptions in the prior year, the number of psychiatry visits and unique prescription types in the prior year, and any history of self-harm.

[4]Multivariable logistic regression adjusting for the same confounders indicated above (3) and for two additional variables; 1) the number of days with an active ACEI or ARB prescription in the 100 days prior to the index date, and 2) the number of days since the most recent active ACEI or ARB prescription day on the index date.

Sensitivity analyses for odds of suicide among those with ARB verses ACEI prescription in the 100 days prior among 2015-2017 VHA users, calculated via conditional logistic regression.

© 2020 Dent KR et al. *JAMA Network Open.*
